# Safety of the Excimer Laser in LASIK and PRK for Patients with Implantable Cardiac Devices: Our Clinical Experience in the Past Two Decades

**DOI:** 10.18502/jovr.v14i4.5473

**Published:** 2019-10-24

**Authors:** Tirth J. Shah, Majid Moshirfar, Phillip C. Hoopes

**Affiliations:** ^1^College of Medicine, University of Arizona, Phoenix, Arizona, USA; ^2^Department of Ophthalmology and Visual Sciences, University of Iowa Hospitals and Clinics, Iowa City, Iowa; ^3^John A. Moran Eye Center, Department of Ophthalmology and Visual Sciences, University of Utah School of Medicine, Salt Lake City, UT, USA; ^4^HDR Research Center, Hoopes Vision, Draper, UT, USA; ^5^Utah Lions Eye Bank, Murray, UT, USA

Sir,

Continued high prevalence in cardiac morbidity in the US has led to more refractive surgical candidates with cardiac implantable electronic devices (CIED), although the Food and Drug Administration (FDA) studies have consistently excluded such populations during laser-assisted *in situ* keratomileusis (LASIK) or photorefractive keratectomy (PRK) evaluations. Major manufacturers have discouraged laser eye surgery in patients with CIEDs until recently when Medtronic and St. Jude, the two largest manufacturers of such devices, approved LASIK surgery with recommendations to shielding the CIED with a magnet and closely monitoring the heart rate during the surgery.^[1]^ We aim to share our insights and experiences over the years, hopefully mitigating some of the concerns in clinical practice.

The excimer laser can electromagnetically interfere with the CIEDs and cause changes in a surgical setting^[2]^ with potential adverse effects including cardiac stimulation inducing ventricular tachycardia or ventricular fibrillation, unexpected movement of the body, and interference with the ability of the CIEDs to adequately monitor for potential arrhythmias.^[3]^ The factors affecting electromagnetic interference depend on the frequency of the emitting device, the distance between the devices, and the amount of shielding of the affected device.^[2]^ Particularly, with excimer use, L'Esperance et al^[4]^ demonstrated that nearly all of the 193 nm energy is absorbed by the cornea indicating that the frequency of light emitted by the ablation process may not cause significant interference with the CIEDs. Furthermore, a recent study by Sher et al^[5]^ demonstrated that *in-vitro* operation with the three most commonly used ophthalmic lasers (VISX Star S4 Excimer Laser, Lumenis Selecta II Glaucoma Laser, and Laserex Ultra Q Photodisruptor) did not lead to oversensing, inappropriate therapy, or change in the programming of the Atlas II+ implantable cardioverter defibrillators (ICDs) or the St. Jude Medical Victory pacemaker.

We retrospectively analyzed data from 1997 to 2014, and found at least 13 patients documented with a CIED, although we expect more patients with such a device who did not disclose this information to us, necessitating the surgeon to ask this important question to all refractive surgery candidates. The average age of patients undergoing LASIK/PRK correction in our center was approximately 35 years (range: 23–60), and in this cohort was 52 years (range: 27–77) with a male predominance (69%) – a consistent finding in individuals with arrhythmia or heart disease. The indications of CIED were primarily for atrial fibrillation (46%), although one male patient aged 27 years had the device for Wolf-Parkinson-White syndrome. The other small subset of patients had the device for either ventricular tachycardia, atrioventricular block of unknown degree, or an unspecified arrhythmia. Out of 13 patients, one patient developed ventricular tachycardia two weeks postoperatively. However, the consulting cardiologist felt the etiology was due to her uncontrolled underlying condition, rather than any electromagnetic interference from the femtosecond and excimer laser that may have offset the device settings. From our records, none of the other patients experienced any CIED-related complications during the surgery. There was no clinically detectable change in the heart rate or rhythm, and postoperatively, the programmed parameters of the pacemakers and/or ICDs remained unchanged. Due to the older age, LASIK enhancement was usually used to achieve emmetropia following the cataract surgery. Thus, we believe this information may be particularly helpful to those clinicians who tend to perform LASIK on the elderly population in their practice.

Each laser and CIED has a unique design and may interfere with one another differently. The current body of evidence and our concomitant experiences illustrate a low risk of complications in patients with CIED during refractive surgery. We believe it is safest to inactivate any anti-tachycardia functions if electrocautery is used during surgery^[3]^ and to follow manufacturer recommendations at all times. With the recent paradigm shift in the advancements to leadless pacemakers, some of these newer devices are immune to electromagnetic interference up to a very high threshold.^[5]^ These remarkable technological advancements will only make ophthalmic lasers safer for patients with CIEDs in the future.

## Financial Support and Sponsorship

This study was funded by an unrestricted Grant from the Research to Prevent Blindness (RPB), 360 Lexington Avenue, 22nd Floor New York, NY 10017. No support was received for the publication of this article.

## Conflicts of Interest

There is no conflict of interest.
